# Psychological Process–Based Profiles in the Binge‐Eating Spectrum: Clinical Implications Beyond DSM‐5 Subtypes

**DOI:** 10.1155/jobe/9309734

**Published:** 2026-10-18

**Authors:** Geovanny Genaro Reivan-Ortiz, María Quezada-Gómez, Laura Maraver-Capdevila, Roser Granero

**Affiliations:** ^1^ Universidad Catolica de Cuenca Cuenca Azuay Ecuador; ^2^ Ministerio de Salud Publica de Ecuador Quito Pichincha Ecuador; ^3^ Department of Psychobiology and Methodology Universitat Autònoma de Barcelona Barcelona 08193 Spain uab.cat

**Keywords:** binge-eating disorder, binge-eating spectrum, bulimia nervosa, cluster, night-eating syndrome

## Abstract

**Background and Objectives:**

Binge‐spectrum eating disorders (BSEDs) are clinically impactful, yet the psychological mechanisms underlying their persistence and heterogeneity remain insufficiently understood. Clarifying these mechanisms is essential for refining diagnostic models and improving treatment. This study focused on young to early‐middle adults, a developmental period with elevated risk for the onset and aggravation of BSED, to identify empirical subgroups based on eating‐ and psychological‐related variables.

**Methods:**

A two‐step cluster analysis was performed in 187 patients (men and women, aged 20–45 years) diagnosed with different BSED subtypes. Variables considered included emotion‐related eating behaviors, impulsivity, emotion dysregulation, decision‐making, stress, anxiety–depression, and obsessive–compulsive symptoms.

**Results:**

A three‐cluster solution provided the best fit, yielding distinct patient profiles. Anxiety–depression and hunger emerged as the most influential variables for the clustering. No significant differences between the clusters were found in sociodemographic characteristics and eating disorder subtype. Clear gradients were observed in clinical severity and psychological functioning, ranging from high psychopathological burden and dysregulation in Cluster 1 (10.2% of the sample) to markedly more adaptive functioning and reduced distress in Cluster 3 (56.7%). The largest between‐cluster differences involved hunger, emotion regulation deficits, and anxiety–depression levels.

**Conclusions:**

The findings delineate clinically meaningful BSED subgroups, spanning severely dysregulated to more adaptive profiles. This differentiation may enhance diagnostic classification and inform process‐based treatment approaches, enabling personalized interventions targeting core features (particularly anxiety–depression, emotion regulation, and hunger) to optimize clinical outcomes.

## 1. Introduction

Binge‐spectrum eating disorders (BSED) refer to a range of eating disorders characterized primarily by recurrent episodes of binge eating, which involve consuming unusually large amounts of food accompanied by a sense of loss of control [1–3]. This spectrum includes disorders, such as binge‐eating disorder (BED), bulimia nervosa (BN), and other specified feeding or eating disorders (OSFED) with binge‐eating features. Night‐eating syndrome (NES) often exhibits overlapping self‐regulatory and neurobehavioral features with binge‐eating presentations, including dysregulated eating patterns, reward‐related eating, and impaired control over food intake. NES can occur across a range of body weight statuses, including individuals with weight within the normative range for age and height. Empirical evidence suggests that NES shares several clinical and psychopathological features with both BED and BN, although it remains a distinct diagnostic entity [4]. But from a transdiagnostic and endophenotypic perspective, NES may be conceptualized within a broader spectrum of binge eating–related conditions, given the partial overlap in mechanisms underlying dysregulated eating behavior.

BSED are associated with significant psychological distress [5, 6], physical health complications [7], and impaired social and occupational functioning [8]. Despite the well‐documented burden associated with BSED, substantial heterogeneity persists in symptom presentation and psychological functioning among affected individuals. Recent research has identified three relatively stable personality and behavioral–psychopathology profiles among individuals exhibiting binge‐eating symptomatology: “Negative Emotionality,” characterized by elevated negative affect, emotion dysregulation, and greater psychiatric comorbidity; “Approach,” associated with heightened reward sensitivity and impulsive/approach‐related behaviors; and “Restrained,” characterized by overcontrol, inhibition, and harm avoidance [9]. Importantly, these profiles exhibit comparable levels of binge‐eating episodes and similar body mass index (BMI), yet differ markedly in their functional characteristics, suggesting meaningful distinctions that extend beyond clinical severity and diagnostic classification. Consequently, elucidating the mechanisms underlying heterogeneity within BSED is essential for refining diagnostic frameworks, identifying clinically relevant subgroups, and informing the development of more personalized treatment approaches.

BSED are increasingly prevalent among younger populations, with emerging and early adulthood (approximately 20–45 years) representing a period of heightened vulnerability. Epidemiological evidence indicates that DSM‐5 eating disorders, including BED, are commonly first identified by early adulthood, with many cases emerging during adolescence and the transition into adult roles [10]. Emerging adulthood is characterized by major life transitions in social roles, living arrangements, and personal responsibilities, which may generate stress and psychological distress that adversely affect well‐being and coping capacities [11, 12]. This developmental period is also marked by key psychosocial transitions, including the formation of intimate partnerships, family establishment, pregnancy, and parenting responsibilities, alongside increasing occupational and caregiving demands. These contextual factors are particularly salient for women and have been consistently associated with increased vulnerability to the onset or exacerbation of eating disorder symptomatology, especially within binge eating–related presentations [13–16]. The convergence of these role transitions and stressors may contribute to increased emotional burden, difficulties in self‐regulation, and reliance on maladaptive coping strategies, thereby elevating risk during this stage of the lifespan [17, 18].

Changes in executive functioning and self‐regulation during early adulthood may also heighten risk for body image concerns and dysregulated eating patterns, as developmentally normative fluctuations in cognitive control and emotion regulation are linked to difficulties managing eating behaviors and negative perceptions of body image [13, 19]. This age range also corresponds to critical periods for weight gain, mood instability, and social stress, which may interact to increase vulnerability to binge‐eating behaviors [20]. The presence of BSED within emerging and early adulthood populations has been estimated primarily among individuals with overweight or obesity, with point prevalences around 8.0% for disordered binge‐spectrum eating and 16.0% for probable subthreshold symptoms [2]. Epidemiological evidence also suggests that the prevalence of BSED has been increasing in recent years [21], reflecting a growing public health concern. This trend underscores the importance of research aimed at identifying underlying vulnerability patterns and mechanisms that could inform early detection and intervention strategies, ultimately helping to prevent the progression and chronicity of these disorders.

Previous research has highlighted the relevance of multiple psychological and behavioral factors in the onset of the BSED, the severity of the clinical profiles, and the treatment outcomes among these patients: emotion (dys)regulation [22–24], impulsivity [25], negative affect [26, 27], stress reactivity [19], decision‐making deficits [28–32], maladaptive eating patterns [3, 33], and compensatory behaviors to influence shape and weight (e.g., purging, driven exercise, and fasting; [34, 35]). Individual differences in eating styles, such as emotional or external eating, further contribute to clinical heterogeneity in individuals within the BSED [36]. Regarding gender, recent evidence suggests that, in clinical samples of individuals seeking treatment for BSED, men and women are more similar than different in their clinical presentation [37].

Impulsivity, a complex multidimensional construct encompassing distinct interacting behavioral and neurocognitive processes, is closely linked to dysfunctions in reward processing and inhibitory control. It has been increasingly recognized as a transdiagnostic vulnerability factor across the heterogeneous conditions encompassed within the BSED [38]. Rather than reflecting a unitary trait, impulsivity comprises multiple facets that differentially contribute to binge‐eating pathology. In particular, negative urgency—the propensity to engage in rash or poorly controlled behaviors in response to heightened negative affect—has been consistently associated with the onset and maintenance of binge‐eating behaviors. This facet is thought to reflect impaired affect‐driven inhibitory control, whereby acute emotional distress amplifies reward sensitivity and weakens top‐down regulatory processes. In addition, attentional impulsivity, characterized by difficulties in sustaining focus and resistance to distraction, and nonplanning impulsivity, reflecting reduced future‐oriented thinking and diminished consideration of long‐term consequences, have been linked to both the presence of binge‐eating behaviors and greater disorder severity [27]. Notably, negative urgency appears to exert a particularly salient influence among individuals with pronounced self‐regulatory deficits and persistent bulimic symptomatology, suggesting its role in reinforcing maladaptive emotion–eating cycles and contributing to the chronicity of bulimic presentations [39].

Neurocognitive studies also support deficits in decision‐making and altered reward sensitivity among individuals with BSED, particularly in response to food cues [32]. Food cue reactivity and craving intensity have been shown to predict binge frequency and the severity of eating disorders across BED and BN [40]. Research on food cue processing and hunger states indicates that individuals with binge‐eating behaviors exhibit greater attentional bias and longer gaze durations toward high‐calorie food stimuli, especially when hungry [41]. Moreover, food craving could act as a transdiagnostic process, mediating the relationship between emotion dysregulation and binge episodes [42]. Interactions between affective–cognitive mechanisms and other physiological systems have been described in BSED, involving dysregulated stress–reward dynamics characterized by heightened responsivity within reward‐related neural circuits—particularly frontostriatal and mesolimbic pathways—concurrent with stress‐induced elevations in negative affect [43]. These alterations are accompanied by deficits in inhibitory control and reward‐based decision‐making, reflecting impaired top‐down executive regulation, which co‐occur with maladaptive eating behaviors and a reduced capacity for craving modulation and resistance to binge‐eating urges [44].

Collectively, these findings suggest that the current diagnostic frameworks provided by widely used taxonomies, such as the *Diagnostic and Statistical Manual of Mental Disorders* (DSM‐5; [45]) or *International Classification of Diseases* (ICD‐11; [46]), may not fully capture meaningful differences in underlying neuropsychological and behavioral processes. While these systems reliably categorize BSED subtypes based on symptom patterns, they overlook the transdiagnostic mechanisms that drive heterogeneity in severity, comorbidity, and functional impairment. This gap underscores the need for empirical approaches, such as clustering modeling, identifying clinically relevant subgroups, and informing more individualized assessment and intervention strategies. However, studies systematically integrating multiple psychological, behavioral, and physiological variables to delineate empirical subgroups among BSED remain limited.

In summary, BMI, hunger, food cue reactivity, impulsivity, emotion regulation, stress, anxiety–depression, and obsessive–compulsive symptoms represent interconnected domains of self‐regulation that cut across traditional diagnostic boundaries in BSED. Rather than viewing these variables as independent features, they can be conceptualized as interacting components of broader transdiagnostic systems underlying binge‐eating pathology. Accordingly, the present study aimed to characterize heterogeneity within a sample of young to early‐middle adults with BSED by applying a data‐driven cluster analysis to a set of clinically and theoretically grounded variables spanning these regulatory domains. The goal was not to reproduce severity gradients or diagnostic categories, but to identify empirically derived subgroups reflecting distinct patterns of covariation across cognitive, emotional, and behavioral systems of self‐regulation. By comparing these subgroups across key clinical and psychological dimensions, this approach seeks to elucidate meaningful profiles of dysfunction that may help explain variability in symptom expression and adaptive functioning beyond traditional categorical frameworks.

## 2. Materials and Methods

### 2.1. Participants

A total of *N* = 187 patients with BSED (41 BN, 35 BED, 30 NES, and 81 OSFED binge‐eating type) were consecutively recruited from the psychiatry outpatient unit of the Citizen “Mental Health” Program, Ministry of Public Health of Ecuador. Participants were aged 18–45 years and had met DSM‐5 criteria for BSED for at least two months before enrollment. Exclusion criteria included: (a) psychoactive substance dependence; (b) medical or neurological conditions causing severe cognitive impairment (e.g., Alzheimer’s disease); (c) current use of psychotropic medication; and (d) being pregnant.

The sample included 119 women (63.64%) and 68 men (36.36%). Most participants were married or lived with a partner (*N* = 121, 64.71%), while *N* = 55 were single (20.41%) and *N* = 11 were divorced‐separated (5.88%).

Table 1 presents the descriptive statistics for the variables analyzed in the study, both for the total sample and stratified by sex. Statistically significant differences between women and men were observed only for hunger and obsessive–compulsive levels, with higher mean scores in women.

**TABLE 1:** Descriptives for the variables of the study for the total sample and stratified by sex.

	Total sample (*N* = 187)		Women (*N* = 119)		Men (*N* = 68)		*p*	CV
	*n*	%	*n*	%	*n*	%		
Marital status							0.390	0.100
Single	55	29.4%	33	27.7%	22	32.4%		
Married—couple	121	64.7%	77	64.7%	44	64.7%		
Divorced—separated	11	5.9%	9	7.6%	2	2.9%		
ED type							0.882	0.060
BN	41	21.9%	26	21.8%	15	22.1%		
BED	35	18.7%	22	18.5%	13	19.1%		
NES	30	16.0%	21	17.6%	9	13.2%		
OSFED	81	43.3%	50	42.0%	31	45.6%		

	**Mean**	**SD**	**Mean**	**SD**	**Mean**	**SD**	**p**	***|d|***

Age (years)	31.82	5.76	32.15	5.97	31.24	5.38	0.297	0.16
BMI (kg/m^2^)	29.83	11.39	29.79	12.57	29.91	9.03	0.944	0.01
ED severity	5.12	2.49	5.12	2.57	5.13	2.37	0.969	0.01
Food cues	16.02	4.24	15.47	4.32	16.99	3.95	0.018	0.37
Food craving	13.93	3.58	13.90	3.87	13.99	3.05	0.875	0.02
Hunger	19.09	6.83	20.01	7.36	17.47	5.45	0.014**^∗^**	0.39
Decision‐making	13.76	6.10	14.40	6.47	12.63	5.25	0.056	0.30
Emotion regulation	12.81	4.50	13.13	4.54	12.25	4.42	0.202	0.20
Impulsiveness	14.64	4.63	14.49	4.74	14.90	4.44	0.562	0.09
Stress	23.78	8.34	24.50	9.10	22.53	6.69	0.121	0.25
Anxiety–depression	14.97	5.40	15.41	5.57	14.19	5.04	0.138	0.23
Obsessive–compulsive	9.31	3.87	9.77	4.16	8.50	3.17	0.030**^∗^**	0.34

*Note:* ED severity: EDI‐3 total. Food cues: PFS total. Food craving: FCQ‐S total. Hunger: TFEQ total. Decision‐making: GDSMS total. Emotion regulation: ERQ total. Impulsiveness: PIS total. Stress: PSS‐10 total. Anxiety–depression: GADS total. Obsessive–compulsive: MOCI total. SD: standard deviation.

Abbreviations: BED, binge‐eating disorder; BMI, body mass index; BN, bulimia nervosa; ED, eating disorder; NES, night‐eating syndrome; OSFED, other specified feeding eating disorder.

^∗^Bold: significant comparison.

### 2.2. Measures

 
*Anthropometry*: BMI was calculated following the standard formula defined by the [47]. Body weight was obtained using a *Health o meter Professional 500KL* digital medical scale (Pelstar, LLC, USA), which has a maximum capacity of 485 kg (500 lb) and is intended for clinical measurement. Participants were assessed while standing upright and facing forward. The collected measurements were then used to compute BMI as follows: BMI = weight (kg)/[height (m)]^2^.
 
*Eating Disorder Severity*: The extent of disordered eating behaviors was evaluated using the *Eating Disorder Inventory-3* (EDI‐3; [48]), a comprehensive self‐report tool developed to measure the severity and presence of psychological and behavioral characteristics linked to eating disorders. This instrument assesses both the core symptoms of eating pathology and the broader psychological factors that influence the onset and persistence of maladaptive eating patterns. The standardized measure includes 91 items rated on a 6‐point Likert scale (0–5) [49–51].
 
*Food Craving*: The momentary intensity of food cravings, understood as a temporary and context‐dependent state, was assessed using the *Brief Form of the Food Craving Questionnaire–State* (FCQ‐S Brief; [52, 53]). This instrument captures the cognitive, emotional, and physiological features that characterize craving episodes. It consists of 10 items rated on a 5‐point Likert scale, producing a total score ranging from 10 to 50. Administration takes approximately 3–5 min. Higher scores indicate a stronger desire or urge to consume palatable foods.
 
*Hunger*: The transient psychological and physiological experience of hunger was evaluated using the *Hunger subscale of the Three-Factor Eating Questionnaire* (TFEQ; [54, 55]). The TFEQ also includes Cognitive Restraint and Uncontrolled Eating/Disinhibition subscales. However, given the strong conceptual and statistical overlap between the Hunger and Uncontrolled Eating/Disinhibition dimensions in binge‐eating populations, only the Hunger subscale was included in the present study to reduce redundancy among clustering variables and avoid the overweighting of highly correlated constructs in the cluster analysis. In addition, the Cognitive Restraint subscale was not included because the study was conducted in individuals within the binge‐eating spectrum, for whom this dimension is considered less central to the clinical phenotype under investigation. The Hunger subscale captures both the intensity and frequency of hunger sensations, as well as the tendency to eat in response to internal hunger cues rather than external or emotional triggers. The self‐report measure includes items rated on a Likert scale (from 1 = “*false*” to 4 = “*true*”), and analyses were conducted using the total score of the Hunger subscale. Higher scores reflect stronger subjective hunger and greater sensitivity to hunger signals.
 
*Food Cues*: Food cue responsiveness was assessed using the *Power of Food Scale* (PFS; [56, 57]). This 15‐item measure assesses the inclination toward “potential consumption,” meaning the desire to eat even in the absence of physical hunger, using a 5‐point Likert response format. The scale captures both motivational and hedonic dimensions of eating, illustrating the degree to which food exerts a psychological “pull” before, during, and after it becomes accessible. The analyses used the raw total score (range = 15–75), with higher values indicating greater vulnerability to the food environment and stronger tendencies toward potential consumption.
 
*Emotion Regulation*: Emotion regulation strategies were assessed using the *Emotion Regulation Questionnaire* (ERQ; [58]). This instrument includes 10 items rated on a 7‐point Likert scale ranging from 1 (*Strongly disagree*) to 7 (*Strongly agree*). In this study, the total score—calculated by summing all item responses—was used as an indicator of the overall application of cognitive emotion regulation strategies [59, 60].
 
*Stress*: Perceived stress was assessed using the 10‐item *Perceived Stress Scale* (PSS‐10; [61, 62]). This widely used self‐report measure evaluates the extent to which individuals view their lives as unpredictable, uncontrollable, and overwhelming. It captures the subjective appraisal of stress experienced over the past month, emphasizing personal evaluations of stressful situations rather than objective stressors. The instrument includes 10 items rated on a 5‐point Likert scale from 0 (*Never*) to 4 (*Very often*). The present study used the total score, where higher values reflect greater perceived stress and reduced perceived coping ability.
 
*Anxiety–Depression*: Symptoms of anxiety and depression were evaluated using the *Goldberg Anxiety and Depression Scale* (GADS; [63, 64]). This self‐report measure consists of 18 dichotomous (yes/no) items divided into two subscales assessing anxiety and depression.
 
*Obsessive–Compulsive*: Obsessive–compulsive symptom severity was evaluated using the Maudsley Obsessive–Compulsive Inventory (MOCI; [65, 66]). This self‐report measure was created to identify and quantify obsessive–compulsive symptoms in both clinical and nonclinical groups, capturing characteristic cognitive and behavioral patterns, such as checking, cleaning, slowness, and doubting. The instrument contains 30 dichotomous items (yes/no), each describing a specific obsessive thought or compulsive action. The total score was used in the analyses, with higher values reflecting more severe obsessive–compulsive symptomatology.
 
*Impulsiveness*: It was measured using the *Plutchik Impulsivity Scale* (PIS; [67, 68]). This self‐report instrument comprises 15 items rated on a Likert scale from 0 (“*never*”) to 3 (“*always*”). The scale evaluates dimensions, such as self‐control, planning, anticipation of consequences, physiological responses, and spontaneous behavior. The total score, obtained by summing all item responses, represents an overall index of impulsivity.
 
*Decision-Making*: Decision‐making tendencies across different situations were assessed using the *General Decision-Making Style* (GDMS; [69]) scale. This instrument views decision‐making as a relatively enduring psychological disposition rather than a response tied to specific circumstances, identifying distinct cognitive and behavioral styles individuals rely on when making choices. The scale comprises 25 statements rated on a 5‐point Likert scale (1 = *strongly disagree* to 5 = *strongly agree*). In this study, the total score was examined as an indicator of overall decision‐making tendencies, with higher scores reflecting greater difficulty or maladaptive patterns in decision‐making [70].
 
*Sociodemographic Variables*: Additional sociodemographic data were collected, including participants’ age, sex, and marital status.

### 2.3. Procedure

The study was conducted in accordance with the most recent version of the Declaration of Helsinki. Approval was granted by both the Research Ethics Committee of the Catholic University of Cuenca and the Clinical Research Ethics Committee of the Ecuadorian Ministry of Public Health (approval code: CEISH‐UCACUE‐2024‐064). All participants provided written informed consent before their inclusion in the study.

Diagnosis of BSED was performed by experienced physicians and psychiatrists specialized in the assessment and treatment of eating disorders. Data collection took place from February to September 2023. To reduce missing responses, clinicians guided participants throughout the completion of all assessment instruments.

### 2.4. Statistical Analysis

Statistical analyses were conducted with SPSS 29 for Windows. Empirical clusters were identified using the TwoStep Cluster procedure. This method was selected for clustering because it is a flexible and robust exploratory technique that allows the identification of homogeneous subgroups within samples, allowing for both categorical and quantitative variables. Although only quantitative variables were included in the present study, this method offers important advantages, such as the automatic estimation of the optimal number of clusters based on statistical fit criteria, thereby reducing subjectivity in cluster selection. In this work, the clustering was performed with the log‐likelihood distance measure, based on the following psychological variables: food cues, food craving, hunger, impulsivity, emotion dysregulation, decision‐making, anxiety–depression, stress, and obsessive–compulsive levels. Variables included in the two‐step cluster analysis were entered in their original metric and were not standardized before model estimation. The optimal cluster solution was selected following Nylund et al. [71], based on (a) the Silhouette coefficient, which assesses cohesion within clusters and separation between clusters [72], with values < 0.30 considered poor, 0.30–0.50 fair, and > 0.50 good; and (b) the clinical interpretability of the clusters.

Differences between clusters were examined with chi‐square tests (*χ* ^2^) and analysis of variance (ANOVA). Multiple testing was controlled using Finner’s method [73]. Effect sizes were calculated with Cramer’s V and Cohen’s d, considering mild–moderate effects as *V* > 0.10 or |*d*| > 0.50, and large effects as *V* > 0.30 or |*d*| > 0.80 [74, 75].

## 3. Results

### 3.1. Clustering

The solutions ranging from 2 to 15 clusters were evaluated (see Table 2). The final three‐cluster solution was selected because it was identified as the optimal solution by the two‐step cluster procedure and provided the most clinically interpretable and parsimonious classification, while maintaining acceptable cluster quality according to the Silhouette coefficient. Figure 1 summarizes the main findings for this selected solution. The bar chart on the left displays the relative importance of the variables used to generate the clusters. The highest contributions corresponded to anxiety–depression level and hunger, followed by emotion regulation, decision‐making, and stress.

**TABLE 2:** Results obtained in the clustering procedure.

Number clusters	BIC index	^a^BIC change	^b^Ratio of BIC changes	AIC index	^a^AIC change	^b^Ratio of AIC changes	^c^Ratio of distance
1	1256.221			1198.061			
2	1040.943	−215.277	1.000	924.623	−273.437	1.000	4.376
3	1064.385	23.441	−0.109	889.905	−34.719	0.127	1.039
4	1090.453	26.068	−0.121	857.813	−32.092	0.117	1.066
5	1120.725	30.273	−0.141	829.926	−27.887	0.102	1.751
6	1178.389	57.664	−0.268	829.430	−0.496	0.002	1.172
7	1241.409	63.020	−0.293	834.290	4.860	−0.018	1.114
8	1307.624	66.215	−0.308	842.345	8.055	−0.029	1.317
9	1380.569	72.944	−0.339	857.129	14.784	−0.054	1.178
10	1456.717	76.149	−0.354	875.118	17.989	−0.066	1.046
11	1533.662	76.945	−0.357	893.902	18.785	−0.069	1.083
12	1611.929	78.267	−0.364	914.010	20.107	−0.074	1.176
13	1692.571	80.641	−0.375	936.491	22.481	−0.082	1.061
14	1773.989	81.418	−0.378	959.750	23.259	−0.085	1.105
15	1856.620	82.631	−0.384	984.221	24.471	−0.089	1.108

*Note:* BIC: Schwarz’s Bayesian criterion.

Abbreviation: AIC, Akaike’s information criterion.

^a^The changes are from the previous number of clusters in the table.

^b^The ratios of changes are relative to the change for the two cluster solutions.

^c^The ratios of distance measures are based on the current number of clusters against the previous number of clusters.

**FIGURE 1:**
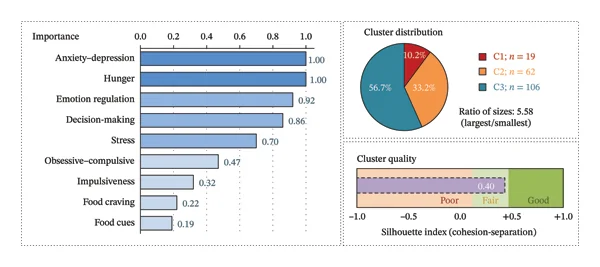
Clustering procedure. *Note.* Food cues: PFS total. Food craving: FCQ‐S total. Hunger: TFEQ total. Decision‐making: GDSMS total. Emotion regulation: ERQ total. Impulsiveness: PIS total. Stress: PSS‐10 total. Anxiety–depression: GADS total. Obsessive–compulsive: MOCI total.

The pie chart in the upper‐right panel shows the distribution of participants across the three identified clusters (C1, C2, and C3), comprising 19 (10.2%), 62 (33.2%), and 106 (56.7%) patients, respectively. This distribution indicated that more than half of the cases were grouped into C3, representing the most prevalent profile in the BSED dataset, while C1 gathered a much smaller fraction of the participants.

The lower‐right panel depicts the cohesion statistic of the clustering solution. The obtained value (0.40) falls within the *fair* range, indicating a mild–moderate level of internal consistency among cluster members.

### 3.2. Comparison Between the Clusters

Table 3 presents the comparison of sociodemographic and clinical variables among the three patient clusters identified in the BSED sample. No significant differences were found between clusters regarding sex, marital status, diagnostic subtype of eating disorder (see also Figure 2), and age. In contrast, pronounced differences emerged in clinical and psychological functioning.

**TABLE 3:** Comparison between the clusters for the variables of the study.

	C1 (*n* = 19)		C2 (*n* = 62)		C3 (*n* = 106)		C1 vs C2		C1 vs C3		C2 vs C3	
	*n*	%	*n*	%	*n*	%	*p*	CV	*p*	CV	*p*	CV
Sex							0.818	0.026	0.186	0.118	0.083	0.134
Women	14	11.76%	44	36.97%	61	51.26%						
Men	5	7.35%	18	26.47%	45	66.18%						
Marital status							0.933	0.042	0.973	0.021	0.806	0.051
Single	6	10.91%	17	30.91%	32	58.18%						
Married—couple	12	9.92%	42	34.71%	67	55.37%						
Divorced—separated	1	9.09%	3	27.27%	7	63.64%						
ED type							0.538	0.164	0.579	0.125	0.570	0.109
BN	4	9.76%	17	41.46%	20	48.78%						
BED	4	11.43%	12	34.29%	19	54.29%						
NES	5	16.67%	8	26.67%	17	56.67%						
OSFED	6	7.41%	25	30.86%	50	61.73%						

	**Mean**	**SD**	**Mean**	**SD**	**Mean**	**SD**	**p**	***|d|***	**p**	***|d|***	**p**	***|d|***

Age (years)	32.79	6.85	32.29	6.06	31.37	5.38	0.947	0.08	0.614	0.23	0.607	0.16
BMI (kg/m^2^)	39.16	16.03	31.76	11.17	27.04	8.69	0.035**^∗^**	**0.54** ^†^	0.001**^∗^**	**0.94** ^†^	0.025**^∗^**	0.47
ED severity	8.68	3.46	5.32	2.28	4.37	1.74	0.001**^∗^**	**1.15** ^†^	0.001**^∗^**	**1.57** ^†^	0.023**^∗^**	0.47
Food cues	18.89	4.14	17.32	3.86	14.75	4.02	0.323	0.39	0.001**^∗^**	**1.02** ^†^	0.001**^∗^**	**0.65** ^†^
Food craving	19.11	3.21	14.24	3.20	12.82	2.98	0.001**^∗^**	**1.52** ^†^	0.001**^∗^**	**2.03** ^†^	0.017**^∗^**	0.46
Hunger	31.42	5.00	22.18	4.88	15.07	3.86	0.001**^∗^**	**1.87** ^†^	0.001**^∗^**	**3.66** ^†^	0.001**^∗^**	**1.62** ^†^
Decision‐making	23.00	6.12	16.73	5.02	10.37	3.54	0.001**^∗^**	**1.12** ^†^	0.001**^∗^**	**2.53** ^†^	0.001**^∗^**	**1.46** ^†^
Emotion regulation	20.26	2.56	14.94	3.81	10.23	2.63	0.001**^∗^**	**1.64** ^†^	0.001**^∗^**	**3.87** ^†^	0.001**^∗^**	**1.44** ^†^
Impulsiveness	20.05	2.80	16.21	4.45	12.75	3.83	0.001**^∗^**	**1.03** ^†^	0.001**^∗^**	**2.18** ^†^	0.001**^∗^**	**0.83** ^†^
Stress	36.21	6.71	28.13	7.26	19.01	4.78	0.001**^∗^**	**1.16** ^†^	0.001**^∗^**	**2.95** ^†^	0.001**^∗^**	**1.48** ^†^
Anxiety–depression	23.11	3.54	17.94	4.71	11.77	3.03	0.001**^∗^**	**1.24** ^†^	0.001**^∗^**	**3.44** ^†^	0.001**^∗^**	**1.56** ^†^
Obsessive–compulsive	13.84	3.59	11.32	3.81	7.32	2.40	0.008**^∗^**	**0.68** ^†^	0.001**^∗^**	**2.13** ^†^	0.001**^∗^**	**1.26** ^†^

*Note:* ED severity: EDI‐3 total. Food cues: PFS total. Food craving: FCQ‐S total. Hunger: TFEQ total. Decision‐making: GDSMS total. Emotion regulation: ERQ total. Impulsiveness: PIS total. Stress: PSS‐10 total. Anxiety–depression: GADS total. Obsessive–compulsive: MOCI total.

Abbreviations: BED, binge‐eating disorder; BMI, body mass index; BN, bulimia nervosa; ED, eating disorder; NES, night‐eating syndrome; OSFED, other specified feeding eating disorder; SD, standard deviation.

^∗^Bold: significant comparison.

^†^Bold: effect size within the mild–moderate to high–large range.

**FIGURE 2:**
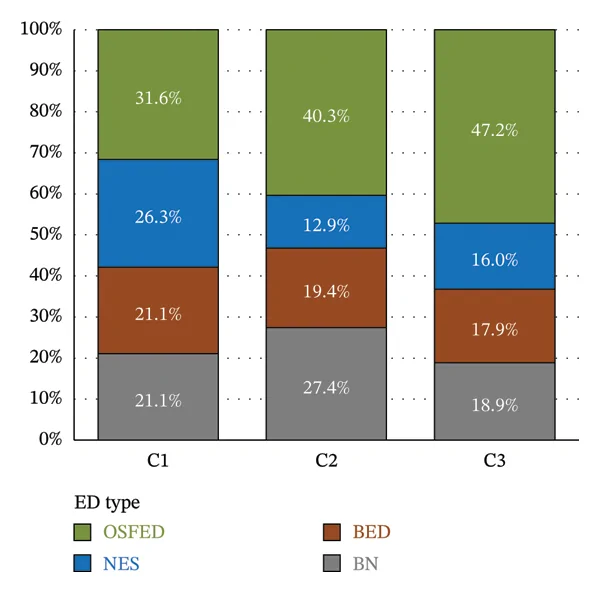
Distribution of eating disorder subtype within clusters. *Note.* ED: eating disorder. BN: bulimia nervosa. BED: binge‐eating disorder. NES: night‐eating syndrome. OSFED: other specified feeding eating disorder.

Patients in C1, which represented the smallest subgroup, displayed the most severe clinical profile. They had the highest BMI and eating disorder severity, along with markedly elevated scores across all psychological dimensions assessed (particularly food craving, hunger, impaired decision‐making, emotion dysregulation, impulsiveness, stress, anxiety–depression, and obsessive–compulsive symptoms). C2 showed intermediate levels of impairment across most domains, while C3 exhibited the most adaptive profile, characterized by lower BMI, reduced eating disorder severity, and the lowest levels of psychological distress. No difference in the mean for food cue reactivity was identified comparing C1 versus C2, but both clusters registered higher means as compared to C3. Effect sizes for the main comparisons ranged from moderate to large, with the strongest differences observed for hunger (|*d*| = 1.87/3.66/1.62), emotion regulation (|*d*| = 1.64/3.87/1.44), and anxiety–depression (|*d*| = 1.24/3.44/1.56).

Figure 3 provides a radar chart summarizing the clinical profiles of the three empirical clusters. The chart illustrates a clear gradient of symptom burden across clusters, ranging from high psychopathological severity and a dysregulated profile in C_1_ (indicative of poor adaptive functioning) to comparatively lower distress and better regulatory functioning in C_3_ (reflecting the most adaptive profile).

**FIGURE 3:**
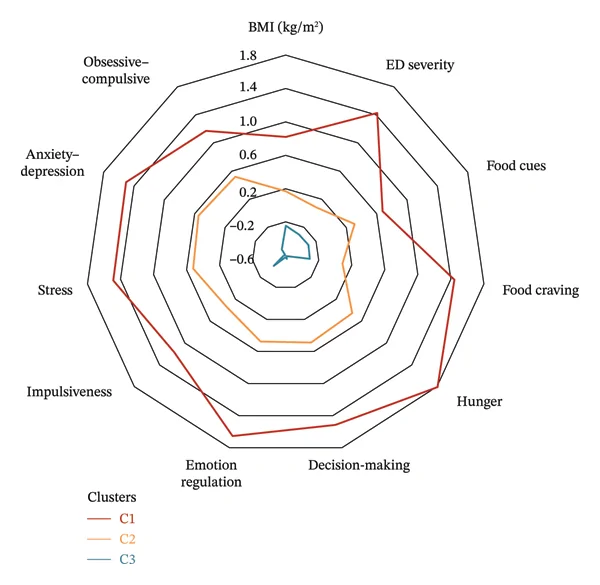
Radar chart. *Note.* BMI: body mass index. ED severity: EDI‐3 total. Food cues: PFS total. Food craving: FCQ‐S total. Hunger: TFEQ total. Decision‐making: GDSMS total. Emotion regulation: ERQ total. Impulsiveness: PIS total. Stress: PSS‐10 total. Anxiety–depression: GADS total. Obsessive–compulsive: MOCI total. Z‐standardized means are plotted.

## 4. Discussion

The present study provides a detailed characterization of heterogeneity among young to early‐middle adults with BSED, revealing three empirically derived clusters that differ markedly in clinical severity, psychological functioning, and regulatory capacities. Although the cluster solution identified three mutually exclusive groups, these clusters should not be interpreted as fully distinct subtypes. Rather, they appear to represent profiles that differ primarily in overall clinical severity, with additional variation across selected behavioral and emotional domains. Anxiety–depression and hunger emerged as the most influential variables in cluster formation, with the largest differences between groups also observed for emotion regulation. These findings underscore the central role of general distress, emotion dysregulation, and physiological cues in distinguishing patient profiles. Moreover, these findings align with prior research emphasizing the role of emotional distress and dysregulated appetite in the maintenance and exacerbation of binge‐spectrum pathology [25, 76, 77].

### 4.1. Psychological Mechanisms Underlying BSED

The analysis of psychological variables indicated that hunger, deficits in emotion regulation, and anxiety–depression showed the largest effect sizes differentiating the identified clusters. From a transdiagnostic perspective, this configuration suggests that heterogeneity in BSED is structured around core dysfunctions spanning multiple RDoC domains, rather than disorder‐specific symptom expressions. In particular, heightened hunger cues point to alterations within the positive valence systems and interoceptive processing, whereas emotion regulation deficits and anxiety–depression map onto the negative valence systems and cognitive systems domains [78]. Within this framework, binge‐spectrum eating behaviors may emerge from dynamic interactions between heightened sensitivity to internal physiological signals and affective vulnerability, combined with reduced regulatory and decision‐making capacities. Consistent with dual‐process and RDoC‐informed models, these interacting domains are likely to bias behavior toward immediate reward or affective relief, especially under conditions of emotional distress. Supporting this interpretation, individuals with binge eating have been shown to exhibit significant impairments in decision‐making [79], as well as elevated impulsivity and choice impulsivity [38, 80], processes that operate transdiagnostically across eating disorders and related forms of psychopathology.

Interestingly, food cue reactivity distinguished C1 and C2 from C3, but did not clearly differentiate between the two higher‐risk clusters when considered in absolute terms. However, a more nuanced interpretation suggests differences in the relative salience of food cue responsiveness within each profile. Specifically, although both clusters showed similarly elevated levels of cue reactivity, this domain appeared to have relatively greater centrality within the overall symptom configuration of C2, whereas C1 was characterized by a broader and more diffuse pattern of elevated dysfunction across multiple domains (including affective distress, impulsivity, and decision‐making difficulties). This pattern suggests that food cue reactivity may play different functional roles across subgroups, being more specifically embedded within an externally driven, cue‐sensitive profile in C2, while representing one component of a more globally dysregulated phenotype in C1. From this perspective, food cue reactivity may reflect not only a general marker of vulnerability to binge‐spectrum pathology, but also a meaningful dimension of heterogeneity in how such vulnerability is expressed. This interpretation is consistent with evidence linking heightened cue reactivity to incentive salience processes and conditioned reward responding, which may differentially interact with broader affective and self‐regulatory mechanisms across individuals. Accordingly, food cue reactivity may contribute to distinct phenotypic expressions within binge‐spectrum presentations, with some individuals showing a more environmentally driven pattern of dysregulated eating and others exhibiting a more pervasive, multidomain profile of psychopathology. This interpretation aligns with prior evidence linking increased cue reactivity to binge‐eating propensity and loss‐of‐control eating, independent of diagnostic status or symptom intensity [77, 81]. From this perspective, food cue reactivity may reflect a shared, early‐emerging process—such as heightened incentive salience or conditioned reward responding—that cuts across binge‐eating presentations, while additional affective and regulatory mechanisms may be required to explain variability among more severe subgroups [82].

Emotion regulation deficits emerged as a particularly salient and discriminative dimension across clusters. Individuals in C1 exhibited markedly greater difficulties in regulating emotions compared to both C2 and C3, suggesting that impaired regulatory capacities may play a central role in amplifying negative affect and precipitating maladaptive binge‐eating behaviors [83]. In this context, difficulties tolerating and modulating emotional distress may increase reliance on binge eating as a short‐term strategy for affective relief, thereby reinforcing loss‐of‐control eating patterns [84]. This interpretation is consistent with evidence indicating that emotion regulation deficits mediate the association between stress exposure and binge eating, highlighting emotion dysregulation as a key transdiagnostic mechanism rather than a mere correlate of symptom severity [85]. Accordingly, the prominence of this dimension in the highest‐risk cluster underscores the potential clinical utility of interventions targeting emotion regulation skills, such as dialectical behavior therapy or other emotion‐focused approaches.

From a developmental perspective, findings related to emotion regulation may also be considered in relation to the examined age range (20–45 years), a period characterized by ongoing changes in cognitive and affective regulatory systems. Recent evidence suggests that individual differences in the maturation of regulatory circuitry during adulthood may confer vulnerability to affective dysregulation and maladaptive coping strategies, potentially exacerbating binge‐spectrum behaviors during this life stage [86, 87]. However, it is important to note that such neurodevelopmental considerations represent only one part of a broader explanatory framework. Sociodemographic and contextual factors (such as educational attainment, employment status, socioeconomic conditions, and changing life roles across adulthood) may also contribute to differences in emotional burden and regulatory capacity between clusters.

### 4.2. Integration With Neurocognitive and Biological Research

From a neurocognitive perspective, the pronounced decision‐making impairments and elevated impulsivity observed in C1 may reflect dysregulation within frontostriatal circuits involved in reward valuation and inhibitory control. This interpretation is consistent with functional neuroimaging evidence showing altered prefrontal engagement during inhibitory control tasks, alongside heightened striatal responsivity to food cues, in individuals with binge eating [79]. Meta‐analytic findings further support this profile, indicating elevated choice impulsivity and steeper delay discounting among individuals with BED compared to controls [38]. Together, these converging lines of evidence suggest that greater BSED severity may be associated with specific neurocognitive vulnerabilities that bias behavior toward immediate reward at the expense of long‐term goals.

Importantly, these neurocognitive alterations are unlikely to operate in isolation. Physiologically, the prominence of hunger as a key differentiating variable highlights the contribution of both homeostatic and hedonic pathways in binge‐spectrum disorders. Dysregulation in appetite‐related signaling—such as altered ghrelin and leptin dynamics—may interact with reward sensitivity and impaired inhibitory control, amplifying binge episodes and contributing to the maintenance of pathological eating patterns [77]. Supporting this integrative view, recent evidence from younger adult samples indicates heightened neural reactivity to hyperpalatable food cues, suggesting that hunger‐related states and cue responsivity may jointly modulate binge risk during early to mid‐adulthood [88].

### 4.3. Implications for Classification and Treatment

Taken together, these findings underscore the clinical value of moving beyond categorical diagnoses and unidimensional severity indicators when conceptualizing and treating BSED. Although BMI and traditional severity markers—such as binge frequency or subtype classification (e.g., BED, BN, NES, and OSFED)—remain clinically informative, they capture only part of the substantial heterogeneity observed within BSED populations. By identifying mechanistically distinct profiles that cut across diagnostic subtypes, our cluster‐based approach complements the DSM‐5 severity specifier [45]—which rely primarily on frequency‐based thresholds—and offers a more nuanced, process‐oriented framework for clinical decision‐making. The present cluster findings suggest that mechanism‐based profiling—encompassing hunger sensitivity, emotion dysregulation, and impulsivity—may provide incremental clinical utility beyond binge frequency alone, complementing prior evidence on the limits of frequency‐based severity classification [89].

More broadly, the observed transdiagnostic configuration of BSED aligns with dimensional models of psychopathology, which emphasize shared mechanisms over categorical labels as targets for intervention [90, 91]. The prominence of affective dysregulation and interoceptive sensitivity across clusters suggests that treatments targeting emotion regulation skills, distress tolerance, and the modulation of hunger and satiety cues may be relevant across diagnostic presentations [92]. Such a mechanism‐focused approach may help account for the heterogeneous treatment responses commonly reported in BSED and supports the development of more personalized intervention strategies.

At the same time, it is important to acknowledge that evidence regarding the relative effectiveness of available interventions for BSED remains limited. Comparative studies are often underpowered, and methodological challenges inherent to psychotherapy trials complicate conclusions regarding the superiority of cognitive–behavioral therapy over alternative approaches, including pharmacological interventions (e.g., fluoxetine, imipramine, desipramine, methylphenidate, and sibutramine) [93]. In this context, the present findings support a move toward stratified treatment planning based on regulatory and neurocognitive profiles rather than diagnosis alone.

Specifically, individuals in C1—representing 10.2% of the sample—exhibited the most severe clinical presentation, characterized by marked affective dysregulation, impaired decision‐making, and elevated impulsivity. This profile is consistent with models positing that binge eating emerges from the interaction between heightened negative affect and compromised self‐control [39]. The substantial psychological burden observed in this cluster suggests the need for intensive, multimodal interventions addressing both affective regulation and executive functioning deficits. Integrative treatments combining cognitive–behavioral strategies with emotion‐focused or dialectical behavior–based approaches may be particularly suitable for this subgroup.

C2 displayed an intermediate level of severity, supporting a dimensional rather than categorical conceptualization of risk. Individuals in this cluster may benefit from moderate‐intensity interventions emphasizing coping strategies, nutritional guidance, and targeted psychological skills to manage binge episodes and associated emotional dysregulation.

By contrast, C3—comprising the majority of the sample (56.7%)—showed relatively preserved adaptive functioning, lower BMI, and reduced symptom severity. For these individuals, maintenance‐oriented approaches focusing on relapse prevention, reinforcement of adaptive coping strategies, and monitoring of risk factors may be most appropriate.

Finally, the age range of the sample (20–45 years) corresponds to early to mid‐adulthood, a developmental period characterized by ongoing plasticity in regulatory and control‐related neural systems. This developmental context further supports the potential benefit of tailoring interventions according to regulatory profiles rather than diagnostic subtypes, as such an approach may optimize treatment responsiveness during this life stage.

### 4.4. Strengths and Limitations

Several strengths and limitations of the present study warrant consideration. Among its strengths, this work provides a comprehensive and multidimensional characterization of heterogeneity within BSED in young to early‐middle adulthood. By integrating clinical, psychological, and regulatory measures, the study identifies empirically derived patient subgroups that cut across traditional diagnostic categories. The inclusion of both men and women, as well as multiple eating disorder subtypes, further enhances the clinical relevance and generalizability of the findings across the broader BSED spectrum.

Several limitations should also be acknowledged. First, the cross‐sectional design precludes causal inferences regarding the relationships among affective, neurocognitive, and physiological processes, and longitudinal studies are needed to determine whether cluster membership predicts treatment response, symptom trajectories, or long‐term outcomes. Second, the sample was restricted to young and early‐middle adults, limiting the generalizability of the results to children, adolescents, or older adult populations. Third, although cluster cohesion fell within the fair range, replication in larger, more diverse samples is necessary to establish the stability and external validity of the identified profiles. Fourth, the operationalization of eating disorder severity was based on the total raw score of the EDI‐3. Since only this global score was available for analysis, with no access to subscale scores, the present study relied on a measure that captures a broader range of psychological characteristics beyond core eating disorder symptomatology. This may limit a more nuanced characterization of the cluster profiles (future research would benefit from the availability of subscale‐level data to more precisely differentiate eating disorder–specific features across subgroups). A further limitation of the present study concerns the restricted availability of sociodemographic and contextual variables that may be relevant to better understanding the pronounced profile observed in the clusters. Although key demographic information was included in the analyses, we did not have access to more detailed indicators, such as socioeconomic status, educational level, employment situation, or family‐related responsibilities, which may contribute to explaining individual differences in symptom severity and cluster membership. Similarly, potential comorbid conditions (e.g., neurodevelopmental or externalizing disorders) that could influence emotion regulation, impulsivity, decision‐making, hunger, and food craving were not assessed in the present sample. Finally, participants were consecutively recruited from a single specialized outpatient mental health unit in Ecuador. Therefore, caution is warranted when generalizing the present findings to other healthcare settings, geographic regions, or community populations with BSED.

Future research should examine the predictive utility of these clusters in clinical contexts, explore the neurobiological and cognitive mechanisms underlying each subgroup, and evaluate the effectiveness of interventions tailored to cluster‐specific vulnerabilities. Integrating psychological, neurocognitive, and biological data may facilitate the development of a precision psychiatry framework for BSED, enabling interventions that maximize therapeutic benefit while minimizing unnecessary treatment intensity. In this regard, a meta‐analysis examining mechanisms of action underlying treatment outcomes in BSED [94] supports the importance of grounding intervention development in empirically supported treatment processes aligned with etiological models. Notably, this work highlights psychological flexibility as a key mechanism associated with symptom reduction, while broader and more sustained improvements in functioning appear linked to gains in emotion regulation, impulse control, and acceptance of negative affect.

## 5. Conclusion

This study identified empirically derived, clinically meaningful subgroups among young to middle‐aged adults with BSED, revealing a spectrum ranging from pronounced dysregulation to relatively preserved adaptive functioning. Overall, the clusters were primarily differentiated along a general severity gradient across most transdiagnostic domains, with anxiety–depression, emotion regulation deficits, and heightened hunger emerging as the most consistent distinguishing features. These findings highlight the central role of emotional distress and dysregulated appetite in the maintenance and exacerbation of binge‐spectrum pathology, while also suggesting that most psychological and behavioral processes assessed tend to covary along a broader severity continuum rather than forming entirely distinct profiles. An exception to this pattern was observed for food cue reactivity, which showed a comparatively different pattern of variation across clusters.

Importantly, the identified clusters transcended traditional diagnostic categories, providing evidence that dimensional and transdiagnostic frameworks more accurately capture the heterogeneity inherent in BSED than categorical classifications alone. Rather than representing purely discrete subtypes, the present findings support a hybrid structure in which an overarching severity dimension coexists with selective domain‐specific differentiation. These findings underscore the value of process‐based approaches in both assessment and intervention, advocating for treatments that target core psychological and physiological mechanisms—such as emotion regulation, interoceptive awareness, and impulsivity—rather than relying solely on diagnostic labels. Ultimately, this framework may inform personalized and mechanism‐driven interventions, improving clinical outcomes across the binge‐spectrum population.

## Author Contributions

Conceptualization: Geovanny Genaro Reivan Ortiz; methodology: Geovanny Genaro Reivan Ortiz and Roser Granero; data collection and management: Geovanny Genaro Reivan Ortiz and María Quezada Gómez; formal analysis: Roser Granero; resources: Geovanny Genaro Reivan Ortiz and Roser Granero; writing–original draft, Geovanny Genaro Reivan Ortiz and Roser Granero; and writing–review and editing: Roser Granero and Laura Maraver‐Capdevila.

## Funding

The authors have nothing to report.

## Disclosure

All authors have read and accepted the published version of the manuscript.

## Ethics Statement

The research was carried out in accordance with the latest version of the Declaration of Helsinki and was approved by the Human Research Ethics Committee of the Catholic University of Cuenca with the code: CEISH–UCACUE–2024‐064 (date: July 07, 2024).

## Consent

Signed consent was obtained from all subjects involved in the study.

## Conflicts of Interest

The authors declare no conflicts of interest.

## Data Availability

The data that support the findings of this study are available from the corresponding author upon reasonable request.

## References

[1] Appolinario J. C. , Sichieri R. , Lopes C. S. et al., Correlates and Impact of DSM-5 Binge Eating Disorder, Bulimia Nervosa and Recurrent Binge Eating: A Representative Population Survey in a Middle-Income Country, Social Psychiatry and Psychiatric Epidemiology. (2022) 57, no. 7, 1491–1503, 10.1007/s00127-022-02223-z. PMC9246799 35044479

[2] Gardizy A. , Lindenfeldar G. , Paul A. , and Chao A. M. , Binge-Spectrum Eating Disorders, Mood, and Food Insecurity in Young Adults With Obesity, Journal of the American Psychiatric Nurses Association. (2024) 30, no. 3, 603–612, 10.1177/10783903221147930. 36600469

[3] Manasse S. M. , Murray H. B. , Parker M. , Forman E. M. , and Juarascio A. S. , A Characterization of Binge Planning Behavior in Individuals With Binge-Spectrum Eating Disorders, Eating and Weight Disorders. (2020) 25, no. 4, 1099–1103, 10.1007/s40519-019-00697-6. 31065976

[4] Kaur J. , Dang A. B. , Gan J. , An Z. , and Krug I. , Night Eating Syndrome in Patients With Obesity and Binge Eating Disorder: A Systematic Review, Frontiers in Psychology. (2022) 12, 10.3389/Fpsyg.2021.766827. PMC8766715 35069340

[5] Gonçalves W. S. , Moraes C. E. F. , Moulin C. C. et al., Exploring Associations Between Suicidal Ideation and Binge Spectrum Eating Disorders: A General Population-Based Survey, Australasian Psychiatry. (2026) 34, no. 1, 47–54, 10.1177/10398562251383783. 41041818

[6] Margaryan L. , Smith K. E. , and Mason T. B. , Daily Patterns of Loneliness and Binge Eating and Food Addiction Using Ecological Momentary Assessment, Journal of Behavioral Medicine. (2025) 48, no. 4, 616–629, 10.1007/s10865-025-00575-w. PMC12307514 40399706

[7] Solmi F. , Moreno A. B. , Lewis G. , Angélica Nunes M. , de Jesus Mendes da Fonseca M. , and Harter Griep R. , Longitudinal Association Between Binge Eating and Metabolic Syndrome in Adults: Findings From the Elsa-Brasil Cohort, Acta Psychiatrica Scandinavica. (2021) 144, no. 5, 464–474, 10.1111/Acps.13356. PMC7613243 34333757

[8] Tabuenca K. , Crowe D. E. , Gillis A. J. et al., Updates in the Treatment of Eating Disorders in 2024: A Year in Review in Eating Disorders: The Journal of Treatment & Prevention, Eating Disorders. (2025) 33, no. 4, 434–450, 10.1080/10640266.2025.2497750. 40340727

[9] Brucar L. R. , Rawls E. , Haynos A. F. , Peterson C. B. , and Zilverstand A. , Mechanism-Based Subtyping in Binge Eating: Understanding Neurobehavioral Heterogeneity Across Negative Emotionality, Approach Behavior, and Executive Function, Translational Psychiatry. (2025) 15, no. 1, 10.1038/s41398-025-03408-1. PMC12144091 40480995

[10] Silén Y. and Keski-Rahkonen A. , Worldwide Prevalence of DSM-5 Eating Disorders Among Young People, Current Opinion in Psychiatry. (2022) 35, no. 6, 362–371, 10.1097/YCO.0000000000000818. 36125216

[11] Galambos N. L. , Krahn H. J. , Johnson M. D. , and Lachman M. E. , The U Shape of Happiness Across the Life Course: Expanding the Discussion, Perspectives on Psychological Science. (2020) 15, no. 4, 898–912, 10.1177/1745691620902428. PMC7529452 32375015

[12] Matud M. P. , Ibáñez I. , Hernández-Lorenzo D. E. , and Bethencourt J. M. , Gender, Life Events, and Mental Well-Being in Emerging Adulthood, International Journal of Social Psychiatry. (2023) 69, no. 6, 1432–1443, 10.1177/00207640231164012. 37029493

[13] Bailey-Straebler S. M. and Susser L. C. , The Impact of Eating Disorders on Reproductive Health: Mitigating the Risk, The Primary Care Companion for CNS Disorders. (2023) 25, no. 4, 10.4088/PCC.22nr03475. 37671825

[14] Call C. C. , Jouppi R. J. , Regal A. L. et al., Eating Disorders and Subthreshold Symptoms in Pregnancy and the Postpartum Period: An Overview of Recent Research and Future Directions for Researchers and Clinicians, Current Psychiatry Reports. (2026) 28, no. 1, 10.1007/s11920-025-01660-2. 41505043

[15] Çiçekoğlu Öztürk P. and Taştekin Ouyaba A. , Prevalence and Related Factors of Eating Disorders in Pregnancy: A Systematic Review and Meta-Analysis, Archives of Gynecology and Obstetrics. (2024) 309, no. 2, 397–411, 10.1007/s00404-023-07051-3. 37162562

[16] Martínez-Olcina M. , Rubio-Arias J. A. , Reche-García C. et al., Eating Disorders in Pregnant and Breastfeeding Women: A Systematic Review, Medicina (Kaunas, Lithuania). (2020) 56, no. 7, 10.3390/Medicina56070352. PMC7404459 32679923

[17] Hormes J. M. , Preconception Weight Suppression Predicts Eating Disorder Symptoms in Pregnancy, European Eating Disorders Review. (2024) 32, no. 4, 633–640, 10.1002/erv.3076. 38361462

[18] Sommerfeldt B. , Skårderud F. , Kvalem I. L. , Gulliksen K. , and Holte A. , Trajectories of Severe Eating Disorders Through Pregnancy and Early Motherhood, Frontiers in Psychiatry. (2024) 14, 10.3389/Fpsyt.2023.1323779. PMC10797066 38250265

[19] Piko B. F. , Kiss H. , Gráczer A. , and Fitzpatrick K. M. , Risk of Disordered Eating in Emerging Adulthood: Media, Body and Weight-Related Correlates Among Hungarian Female University Students, Journal of Preventive Medicine and Hygiene. (2022) 63, no. 1, E83–E89, 10.15167/2421-4248/Jpmh2022.63.1.2375. PMC9121682 35647372

[20] Kosmas J. A. , Wildes J. E. , Graham A. K. , and O’connor S. M. , The Role of Stress in the Association Among Food Insecurity, Eating Disorder Pathology, and Binge Eating-Related Appetitive Traits, Eating Behaviors. (2023) 49, 10.1016/j.Eatbeh.2023.101709. PMC10238633 36822033

[21] Liu K. , Gao R. , Kuang H. , E R. , Zhang C. , and Guo X. , Global, Regional, and National Burdens of Eating Disorders in Adolescents and Young Adults Aged 10-24 Years From 1990 to 2021: A Trend Analysis, Journal of Affective Disorders. (2025) 388, 10.1016/j.jad.2025.119596. 40480381

[22] Riddle D. R. , Presseller E. K. , Manasse S. M. , and Juarascio A. S. , Latent Profiles of Emotion Dysregulation Among Individuals With Binge-Spectrum Eating Disorders: Associations With Eating Disorder Pathology, European Eating Disorders Review. (2023) 31, no. 6, 793–801, 10.1002/erv.3009. PMC10592055 37394985

[23] Walenda A. , Bogusz K. , Kopera M. , Jakubczyk A. , Wojnar M. , and Kucharska K. , Emotion Regulation in Binge Eating Disorder, Psychiatria Polska. (2021) 55, no. 6, 1433–1448, 10.12740/PP/Onlinefirst/122212. 35472237

[24] Yang H. , Zhou X. , Xie L. , and Sun J. , The Effect of Emotion Regulation on Emotional Eating Among Undergraduate Students in China: The Chain Mediating Role of Impulsivity and Depressive Symptoms, Plos One. (2023) 18, no. 6, 10.1371/Journal.Pone.0280701. PMC10270349 37319167

[25] Wilkinson M. L. and Juarascio A. S. , Heterogeneous Profiles of Impulsivity are Associated With Clinical Severity and Treatment Outcomes Among Adults With Binge-Spectrum Eating Disorders, Eating Disorders. (2025) 1–13, 1–13, 10.1080/10640266.2025.2520563. PMC12354265 40568875

[26] Hill D. , Conner M. , Clancy F. et al., Stress and Eating Behaviours in Healthy Adults: A Systematic Review and Meta-Analysis, Health Psychology Review. (2022) 16, no. 2, 280–304, 10.1080/17437199.2021.1923406. 33913377

[27] Lampe E. W. , Crochiere R. J. , Moussaoui J. R. et al., Negative Affect Mediates the Association Between Nightly Sleep Disturbance and Next-Day Binge Eating, Appetite. (2025) 216, 10.1016/j.Appet.2025.108261. PMC13129479 40782833

[28] Mallorquí-Bagué N. , Lozano-Madrid M. , Granero R. et al., Cognitive and Clinical Gender-Related Differences Among Binge-Spectrum Eating Disorders: Analysis of Therapy Response Predictors, European Eating Disorders Review. (2023) 31, no. 3, 377–389, 10.1002/erv.2961. 36482806

[29] Munguía L. , Lucas I. , Jiménez-Murcia S. et al., Executive Functions in Binge Spectrum Eating Disorders With Comorbid Compulsive Buying, European Eating Disorders Review. (2021) 29, no. 6, 854–867, 10.1002/erv.2855. 34322952

[30] Reiter A. M. , Heinze H. J. , Schlagenhauf F. , and Deserno L. , Impaired Flexible Reward-Based Decision-Making in Binge Eating Disorder: Evidence From Computational Modeling and Functional Neuroimaging, Neuropsychopharmacology. (2017) 42, no. 3, 628–637, 10.1038/npp.2016.95. PMC5240187 27301429

[31] Testa G. , Granero R. , Misiolek A. et al., Impact of Impulsivity and Therapy Response in Eating Disorders From a Neurophysiological, Personality and Cognitive Perspective, Nutrients. (2022) 14, no. 23, 10.3390/nu14235011. PMC9738347 36501041

[32] Vajdová D. , Ježková J. , Procházková P. , Roubalová R. , and Patrono E. , Evidence for Food-Related and non-Food-Related Maladaptive Preference in a Mouse Model of Binge Eating Disorder, Frontiers in Behavioral Neuroscience. (2025) 19, 10.3389/Fnbeh.2025.1653807. PMC12580334 41190266

[33] Presseller E. K. , Lampe E. W. , Nunez N. , and Juarascio A. S. , Latent Profiles of Dietary Restraint Among Individuals With Binge-Spectrum Eating Disorders: Associations With Eating Disorder Symptom Severity, International Journal of Eating Disorders. (2022) 55, no. 12, 1843–1852, 10.1002/eat.23816. PMC9742196 36161726

[34] Srivastava P. , Lampe E. W. , Wons O. B. , Juarascio A. S. , and Manasse S. M. , Understanding Momentary Associations Between Body Dissatisfaction and Exercise in Binge-Spectrum Eating Disorders, Eating and Weight Disorders. (2022) 27, no. 6, 2193–2200, 10.1007/s40519-022-01371-0. PMC13112405 35122639

[35] Wilkinson M. L. , Presseller E. K. , Lampe E. W. et al., The Relationship Between Non-Purging Compensatory Behaviors, Clinical Severity, and Treatment Outcomes in Adults With Binge-Spectrum Eating Disorders, Eating Disorders. (2024) 32, no. 2, 212–222, 10.1080/10640266.2023.2293504. PMC10922548 38186089

[36] Benbaibeche H. , Saidi H. , Bounihi A. , and Koceir E. A. , Emotional and External Eating Styles Associated With Obesity, Journal of Eating Disorders. (2023) 11, no. 1, 10.1186/s40337-023-00797-w. PMC10152697 37131270

[37] Sonnenblick R. M. and Juarascio A. S. , Men and Women Who Seek Treatment for Binge-Spectrum Eating Disorders Have Similar Baseline Characteristics, Appetite. (2026) 216, 10.1016/j.Appet.2025.108254. PMC13261554 40752787

[38] Carr M. M. , Wiedemann A. A. , Macdonald-Gagnon G. , and Potenza M. N. , Impulsivity and Compulsivity in Binge Eating Disorder: A Systematic Review of Behavioral Studies, Progress in Neuro-Psychopharmacology & Biological Psychiatry. (2021) 110, 10.1016/j.Pnpbp.2021.110318. PMC8222068 33794320

[39] Forester G. , Wonderlich J. A. , Bottera A. R. et al., Behaviourally Assessed Negative Urgency is Uniquely Associated With Binge-Eating Frequency, European Eating Disorders Review. (2025) 33, no. 1, 3–9, 10.1002/erv.3124. PMC11620958 38977861

[40] Schaefer L. M. , Forester G. , Burr E. K. et al., Examining the Role of Craving in Affect Regulation Models of Binge Eating: Evidence From an Ecological Momentary Assessment Study, Journal of Psychopathology and Clinical Science. (2023) 132, no. 6, 725–732, 10.1037/abn0000839. PMC10695167 37307313

[41] Woo J. M. , Lee G. E. , and Lee J. H. , Attentional Bias for High-Calorie Food Cues by the Level of Hunger and Satiety in Individuals With Binge Eating Behaviors, Frontiers in Neuroscience. (2023) 17, 10.3389/Fnins.2023.1149864. PMC10372423 37521694

[42] Lopez-Guerrero J. , Navas J. F. , Perales J. C. , Rivero F. J. , and Muela I. , The Interrelation Between Emotional Impulsivity, Craving, and Symptoms Severity in Behavioral Addictions and Related Conditions: A Theory-Driven Systematic Review, Current Addiction Reports. (2023) 10, no. 4, 718–736, 10.1007/s40429-023-00512-4.

[43] Donnelly B. , Touyz S. , Hay P. , Burton A. , Russell J. , and Caterson I. , Neuroimaging in Bulimia Nervosa and Binge Eating Disorder: A Systematic Review, Journal of Eating Disorders. (2018) 6, no. 1, 10.1186/s40337-018-0187-1. PMC5819247 29468065

[44] Pasquale E. K. , Boyar A. M. , and Boutelle K. N. , Reward and Inhibitory Control as Mechanisms and Treatment Targets for Binge Eating Disorder, Current Psychiatry Reports. (2024) 26, no. 11, 616–625, 10.1007/s11920-024-01534-z. PMC11579074 39316228

[45] American Psychiatric Association , Diagnostic and Statistical Manual of Mental Disorders, 2013, 5th edition, American Psychiatric Publishing, Arlington, VA.

[46] World Health Organization , International Classification of Diseases for Mortality and Morbidity Statistics (11th Rev.), 2022, WHO, https://icd.who.int/browse/2025-01/mms/en.

[47] World Health Organization , Obesity: Preventing and Managing the Global Epidemic. Report of a WHO Consultation (WHO Technical Report Series No. 894), 2000, World Health Organization. 11234459

[48] Garner D. M. and Van Strien T. , EDI-3. Eating Disorder Inventory-3: Professional Manual, 2004, https://www.hogrefe.nl/shop/media/downloads/sample-reports/5702512_InkijkexemplaarHandleiding_Samplepages.pdf.

[49] Clausen L. , Rosenvinge J. H. , Friborg O. , and Rokkedal K. , Validating the Eating Disorder Inventory-3 (EDI-3): A Comparison Between 561 Female Eating Disorders Patients and 878 Females From the General Population, Journal of Psychopathology and Behavioral Assessment. (2011) 33, no. 1, 101–110, 10.1007/s10862-010-9207-4. PMC3044826 21472023

[50] Goldschmidt A. B. , Wall M. M. , Zhang J. , Loth K. A. , and Neumark-Sztainer D. , Overeating and Binge Eating in Emerging Adulthood: 10-Year Stability and Risk Factors, Developmental Psychology. (2016) 52, no. 3, 475–483, 10.1037/dev0000086. PMC4760881 26689758

[51] Reivan O. G. G. , Campoverde X. , Vinañzaca J. , Estrada J. , Yanza R. , and Granero R. , Factors Increasing the Risk for Food Addiction in Ecuadorian Students, Frontiers in Psychiatry. (2024) 14, 10.3389/Fpsyt.2023.1214266. PMC10792013 38234363

[52] Cepeda-Benito A. , Gleaves D. H. , Fernández M. C. , Vila J. , Williams T. L. , and Reynoso J. , The Development and Validation of Spanish Versions of the State and Trait Food-Cravings Questionnaires, Behaviour Research and Therapy. (2000) 38, no. 11, 1125–1138, 10.1016/S0005-7967(99)00141-2. 11060941

[53] Cepeda-Benito A. , Gleaves D. H. , Williams T. L. , and Erath S. A. , The Development and Validation of the State and Trait Food-Cravings Questionnaires, Behavior Therapy. (2000) 31, no. 1, 151–173, 10.1016/S0005-7894(00)80009-X. 11060941

[54] Jáuregui-Lobera I. , García-Cruz P. , Carbonero-Carreño R. , Magallares A. , and Ruiz-Prieto I. , Psychometric Properties of Spanish Version of the Three-Factor Eating Questionnaire-R18 (Tfeq-Sp), Nutrients. (2014) 6, no. 12, 5619–5635, 10.3390/nu6125619. PMC4276988 25486370

[55] Stunkard A. J. and Messick S. , The Three-Factor Eating Questionnaire to Measure Dietary Restraint, Disinhibition and Hunger, Journal of Psychosomatic Research. (1985) 29, no. 1, 71–83, 10.1016/0022-3999(85)90010-8. 3981480

[56] Lowe M. R. , Butryn M. L. , Didie E. R. et al., The Power of Food Scale. A New Measure of the Psychological Influence of the Food Environment, Appetite. (2009) 53, no. 1, 114–118, 10.1016/j.Appet.2009.05.016. 19500623

[57] Serier K. N. , Belon K. E. , Smith J. M. , and Smith J. E. , Psychometric Evaluation of the Power of Food Scale in a Diverse College Sample: Measurement Invariance Across Gender, Ethnicity, and Weight Status, Eating Behaviors. (2019) 35, 10.1016/j.Eatbeh.2019.101336. 31731234

[58] Gross J. J. and John O. P. , Individual Differences in Two Emotion Regulation Processes: Implications for Affect, Relationships, and Well-Being, Journal of Personality and Social Psychology. (2003) 85, no. 2, 348–362, 10.1037/0022-3514.85.2.348. 12916575

[59] Cabello R. , Salguero J. M. , Fernández-Berrocal P. , and Gross J. J. , A Spanish Adaptation of the Emotion Regulation Questionnaire, European Journal of Psychological Assessment. (2013) 29, no. 4, 234–240, 10.1027/1015-5759/a000150.

[60] Moreta-Herrera R. , Perdomo-Pérez M. , Vaca-Quintana D. et al., New Psychometric Evidence of a Bifactor Structure of the Emotion Regulation Questionnaire (ERQ) in Ecuadorian College Students, Psychology in Russia: State of the Art. (2022) 15, no. 1, 120–134, 10.11621/pir.2022.0108. PMC9845005 36699814

[61] Cohen S. , Kamarck T. , and Mermelstein R. , A Global Measure of Perceived Stress, Journal of Health and Social Behavior. (1983) 24, no. 4, 385–396, 10.2307/2136404. 6668417

[62] Ruisoto P. , López-Guerra V. M. , Paladines M. B. , Vaca S. L. , and Cacho R. , Psychometric Properties of the Three Versions of the Perceived Stress Scale in Ecuador, Physiology & Behavior. (2020) 224, 10.1016/j.Physbeh.2020.113045. 32619529

[63] Goldberg D. , Bridges K. , Duncan-Jones P. , and Grayson D. , Detecting Anxiety and Depression in General Medical Settings, BMJ. (1988) 297, no. 6653, 897–899, 10.1136/bmj.297.6653.897. PMC1834427 3140969

[64] Reivan-Ortiz G. , Pineda-Garcia G. , and León B. , Psychometric Properties of the Goldberg Anxiety and Depression Scale (Gads) in an Ecuadorian Population, International Journal of Psychological Research. (2019) 12, no. 1, 41–48, 10.21500/20112084.3745. PMC7110168 32612786

[65] Hodgson R. J. and Rachman S. , Obsessional-Compulsive Complaints, Behaviour Research and Therapy. (1977) 15, no. 5, 389–395, 10.1016/0005-7967(77)90042-0. 612339

[66] Ávila-Espada A. , Una Contribución a La Evaluación De Las Obsesiones Y Compulsiones: Revisión Del Inventario De Obsesiones De Leyton (LOI) Y Del Cuestionario Obsesivo-Compulsivo De Maudsley (Mocq), Psiquis. (1986) 7, 93–99, https://www.psicopsi.com/obsesivo-compulsivo-de-maudsley/.

[67] Plutchik R. and Van Praag H. M. , The Measurement of Suicidality, Aggressivity and Impulsivity, Progress in Neuro-Psychopharmacology and Biological Psychiatry. (1989) 13, no. 4–5, 523–534, 10.1016/0278-5846(89)90107-3. 2616792

[68] Rubio G. , Montero I. , Jáuregui J. et al., Validación De La Escala De Impulsividad De Plutchik En Población Española, Archivos de Neurobiologia. (1998) 61, no. 4, 223–232, 10.21865/RideP54.1.12.

[69] Scott S. G. and Bruce R. A. , Decision-Making Style: The Development and Assessment of a New Measure, Educational and Psychological Measurement. (1995) 55, no. 5, 818–831, 10.1177/0013164495055005017.

[70] Alacreu A. , Fuentes M. , Abad D. , Cano I. , González E. , and Serrano M. , Spanish Validation of the General Decision-Making Style Scale: Sex Invariance, Sex Differences, and Relationships With Personality and Coping Styles, Judgment and Decision Making. (2019) 14, no. 6, 739–751, 10.1017/S1930297500005453.

[71] Nylund K. L. , Asparouhov T. , and Muthén B. O. , Deciding on the Number of Classes in Latent Class Analysis and Growth Mixture Modeling: A Monte Carlo Simulation Study, Structural Equation Modeling. (2007) 14, no. 4, 535–569, 10.1080/10705510701575396.

[72] Rousseeuw P. J. , Silhouettes: A Graphical Aid to the Interpretation and Validation of Cluster Analysis, Journal of Computational and Applied Mathematics. (1987) 20, 53–65, 10.1016/0377-0427(87)90125-7.

[73] Finner H. and Roters M. , On the False Discovery Rate and Expected Type I Errors, Biometrical Journal. (2001) 43, no. 8, 985–1005.

[74] Cohen J. , Statistical Power Analysis for the Behavioral Sciences, 1988, 2nd edition, Lawrence Erlbaum Associates, Publishers, Hillsdale, NJ.

[75] Kelley K. and Preacher K. J. , On Effect Size, Psychological Methods. (2012) 17, no. 2, 137–152, 10.1037/a0028086. 22545595

[76] Giel K. E. , Bulik C. M. , Fernandez-Aranda F. et al., Binge Eating Disorder, Nature Reviews Disease Primers. (2022) 8, no. 1, 10.1038/s41572-022-00344-y. PMC9793802 35301358

[77] Musquez M. and Rasmussen E. B. , Food Cue Reactivity Meets the Reinforcer Pathology Model: Behavioral Economic Measures of cue-Induced Changes in Food Reinforcer Efficacy, Perspectives on Behavior Science. (2024) 47, no. 2, 393–416, 10.1007/s40614-024-00409-1. PMC11294300 39099745

[78] Leenaerts N. , Jongen D. , Ceccarini J. , Van Oudenhove L. , and Vrieze E. , The Neurobiological Reward System and Binge Eating: A Critical Systematic Review of Neuroimaging Studies. the International *Journal of Eating Disorders* , International Journal of Eating Disorders. (2022) 55, no. 11, 1421–1458, 10.1002/eat.23776. 35841198

[79] Colton E. , Wilson K. E. , Chong T. T. J. , and Verdejo-Garcia A. , Dysfunctional Decision-Making in Binge-Eating Disorder: A Meta-Analysis and Systematic Review, Neuroscience & Biobehavioral Reviews. (2023) 152, 10.1016/j.Neubiorev.2023.105250. 37263530

[80] Yan W. S. , Zheng D. H. , and Liu M. M. , Trait Impulsivity and Choice Impulsivity in Young Adult Students With Probable Binge Eating Disorder, Frontiers in Psychiatry. (2022) 13. 10.3389/fpsyt.2022.838700 PMC9037330 35479492

[81] Meule A. , Küppers C. , Harms L. et al., Food Cue-Induced Craving in Individuals With Bulimia Nervosa and Binge-Eating Disorder, Plos One. (2018) 13, no. 5, 10.1371/Journal.Pone.0204151. PMC6136823 30212574

[82] Vrieze E. and Leenaerts N. , Neuronal Activity and Reward Processing in Relation to Binge Eating, Current Opinion in Psychiatry. (2023) 36, no. 6, 443–448, 10.1097/YCO.0000000000000895. 37781973

[83] da Luz F. Q. , Mohsin M. , Jana T. A. et al., An Examination of the Relationships Between Eating-Disorder Symptoms, Difficulties With Emotion Regulation, and Mental Health in People With Binge Eating Disorder, Behavioral Sciences. (2023) 13, no. 3, 10.3390/bs13030234. PMC10045385 36975259

[84] Borm I. M. , Hartmann S. , Barnow S. , and Pruessner L. , Binge Eating as Emotion Regulation? A Meta-Analysis of Ecological Momentary Assessment Studies, Clinical Psychology Review. (2025) 121, 10.1016/j.cpr.2025.102625. 40768884

[85] Schnepper R. , Reichenberger J. , and Blechert J. , ‘Bad Mood Food?’ Increased Versus Decreased Food Cue-Reactivity in Emotional Contexts in Bulimia Nervosa, European Eating Disorders Review. (2021) 29, no. 6, 847–858, 10.1002/erv.2849. 34176193

[86] Sonnenblick R. M. , Williams E. A. , Lavender J. M. , and Manasse S. M. , Good for Men, but Not for Me: A Qualitative Study of Factors Preventing Men From Seeking Psychological Treatment for Their Binge-Spectrum Eating Disorders, Psychology of Men & Masculinities. (2026) 27, no. 1, 60–68, 10.1037/men0000543.

[87] Whittle S. , Zhang L. , and Rakesh D. , Environmental and Neurodevelopmental Contributors to Youth Mental Illness, Neuropsychopharmacology. (2025) 50, no. 1, 201–210, 10.1038/s41386-024-01926-y. PMC11526094 39030435

[88] Hiluy J. C. , David I. A. , Lobo I. et al., Altered Brain Reactivity to Food Cues in Undergraduate Students With Disordered Eating Behaviours, Biomedicines. (2024) 12, no. 8, 10.3390/Biomedicines12081656. PMC11351394 39200121

[89] Dakanalis A. , Colmegna F. , Riva G. , and Clerici M. , Validity and Utility of the DSM‐5 Severity Specifier for Binge‐Eating Disorder, International Journal of Eating Disorders. (2017) 50, no. 8, 917–923, 10.1002/eat.22696. 28245061

[90] Berking M. , Eichler E. , Naumann E. , and Svaldi J. , The Efficacy of a Transdiagnostic Emotion Regulation Skills Training in the Treatment of Binge-Eating Disorder-Results From a Randomized Controlled Trial, British Journal of Clinical Psychology. (2022) 61, no. 4, 998–1018, 10.1111/bjc.12371. 35567309

[91] Rodrigues T. F. and Machado P. P. P. , Emotion Regulation and Eating Disorders—The Current Status of Research and Future Directions, Current Opinion in Psychiatry. (2025) 38, no. 6, 421–426, 10.1097/YCO.0000000000001043. 40956317

[92] Poovey K. , Ahlich E. , Attaway S. , and Rancourt D. , General Versus Hunger/Satiety-Specific Interoceptive Sensibility in Predicting Disordered Eating, Appetite. (2022) 171, 10.1016/j.Appet.2022.105930. 35033582

[93] Samara M. T. , Michou N. , Lappas A. S. et al., Is Cognitive Behavioral Therapy More Effective Than Pharmacotherapy for Binge Spectrum Disorders? A Systematic Review and Meta-Analysis, Australian and New Zealand Journal of Psychiatry. (2024) 58, no. 4, 308–319, 10.1177/00048674231219593. 38179705

[94] Barney J. L. , Murray H. B. , Manasse S. M. , Dochat C. , and Juarascio A. S. , Mechanisms and Moderators in Mindfulness- and Acceptance-Based Treatments for Binge Eating Spectrum Disorders: A Systematic Review, European Eating Disorders Review. (2019) 27, no. 4, 352–380, 10.1002/erv.2673. PMC6570825 30887695

